# An Efficient *Agrobacterium rhizogenes*-Mediated Hairy Root Transformation Method in a Soybean Root Biology Study

**DOI:** 10.3390/ijms232012261

**Published:** 2022-10-14

**Authors:** Penghui Huang, Mingyang Lu, Xiangbei Li, Huiyu Sun, Zhiyuan Cheng, Yuchen Miao, Yongfu Fu, Xiaomei Zhang

**Affiliations:** 1 Moa Key Lab of Soybean Biology (Beijing), National Key Facility of Crop Gene Resource and Genetic Improvement, Institute of Crop Sciences, Chinese Academy of Agricultural Sciences, Beijing 100081, China; 2 The Key Laboratory of Plant Resources Conservation and Germplasm Innovation in the Mountainous Region (Ministry of Education), Institute of Agro-Bioengineering, Guizhou University, Guiyang 550025, China; 3 College of Agronomy, Sichuan Agricultural University, Chengdu 611130, China; 4 CAS Key Laboratory of Soybean Molecular Design Breeding, Northeast Institute of Geography and Agroecology, Chinese Academy of Sciences, Changchun 130102, China; 5 State Key Laboratory of Crop Stress Adaptation and Improvement, School of Life Sciences, Henan University, Kaifeng 475004, China

**Keywords:** hairy root regeneration and transformation, nodulation, *Agrobacterium*, *GmNSP1*, GmNup107-160 sub-complex, promoter analysis, soybean

## Abstract

The stable genetic transformation of soybean is time-consuming and inefficient. As a simple and practical alternative method, hairy root transformation mediated by *Agrobacterium rhizogenes* is widely applied in studying root-specific processes, nodulation, biochemical and molecular functions of genes of interest, gene editing efficiency of CRISPR/Cas9, and biological reactors and producers. Therefore, many laboratories have developed unique protocols to obtain hairy roots in composite plants composed of transgenic roots and wild-type shoots. However, these protocols still suffer from the shortcomings of low efficiency and time, space, and cost consumption. To address this issue, we developed a new protocol efficient regeneration and transformation of hairy roots (eR&T) in soybean, by integrating and optimizing the main current methods to achieve high efficiency in both hairy root regeneration and transformation within a shorter period and using less space. By this eR&T method, we obtained 100% regeneration of hairy roots for all explants, with an average 63.7% of transformation frequency, which promoted the simultaneous and comparative analysis of the function of several genes. The eR&T was experimentally verified *Promoter:GUS* reporters, protein subcellular localization, and CRISPR/Cas9 gene editing experiments. Employing this approach, we identified several novel potential regulators of nodulation, and nucleoporins of the Nup107-160 sub-complex, which showed development-dependent and tissue-dependent expression patterns, indicating their important roles in nodulation in soybean. Thus, the new eR&T method is an efficient and economical approach for investigating not only root and nodule biology, but also gene function.

## 1. Introduction

Soybean (*Glycine max* (L.) Merr.), one of the most widely grown legumes in the world, is a major source of plant proteins and oils for humans and animals [1,2,3]. Benefiting from simple operation, good repeatability, low experimental cost, and stabilization of exogenous genes in transgenic lines, the *Agrobacterium tumefaciens*-mediated transgenic method has become the best choice for transforming plants, including soybean. Although a foreign gene can be stably integrated into the genome of soybean in this way [4], the traditional *Agrobacterium tumefaciens*-mediated stable soybean transformation method, with low efficiency and long duration, presents shortcomings for study of soybean gene function [4,5]. Compared with the stable transformation mediated by *Agrobacterium tumefaciens*, hairy root transformation mediated by *Agrobacterium rhizogenes* is faster and easier to conduct. Hairy root transformation systems have been established in soybean to study drought stress, pathogen infection, and nitrogen fixation symbiosis [4,5,6,7,8,9]. Current methods of hairy root transformation can be roughly classed into two groups. (1) Direct plant inoculation [6,10] in which *Agrobacterium rhizogenes* liquid is directly injected with a syringe to the hypocotyl of plant seedlings. After a period of culture, hairy roots are produced at the injection site. This one-step method [5,11] involves selecting sterile plant seedlings, cutting plant hypocotyls obliquely at an early stage of plant development, injuring them and soaking them in a suspension of *Agrobacterium rhizogenes*. The injured seedlings are planted back into the soil or grown on germination paper, then hairy roots are produced at the incision site after a period of culture. (2) The explant inoculation method [12] involves sterile seedlings of plants being co-cultured with *Agrobacterium rhizogenes*, sub-cultured on selected medium, with transformed cells producing callus and inducing hairy roots. However, these protocols have limitations such as long duration and significant space consumption; they also have low efficiency with a limited number of transgenic hairy roots produced. The methods of Kereszt et al. [6] and Fan et al. [5] are not only inefficient but also time-consuming. The Song et al. method [11] is simple and time-saving, but the transformation efficiency and transformation frequency are relatively low, whereas a method derived by Cheng et al. [12] had higher efficiency of transformation, but the positive hairy roots were not suitable for subsequent nodulation analysis, since they used 2/3 sections of cotyledons without embryo as explants, which could not produce shoots later in development.

In this study, we report an efficient regeneration and transformation method for hairy roots (eR&T) in soybean by integrating and optimizing the main, current methods to gain high efficiency in both hairy root regeneration and transformation within a shorter period and using less space. The method takes only 18 days for the eR&T process to produce composite plants, composed of transgenic roots and wild-type shoots, with a 100% regeneration rate and 63.7% of transformation frequency. The eR&T method is reliable and feasible for gene functional analyses and biotechnological applications in soybean, including *Promoter:GUS* reporters, protein subcellular localization, and CRISPR/Cas9 gene editing. We also identified some novel genes involved in nodulation in soybean, such as *GmNup107* and *GmNup160*, with our eR&T approach.

## 2. Results

### 2.1. Optimization of Soybean Hairy Root Transformation

**Figure 1:**
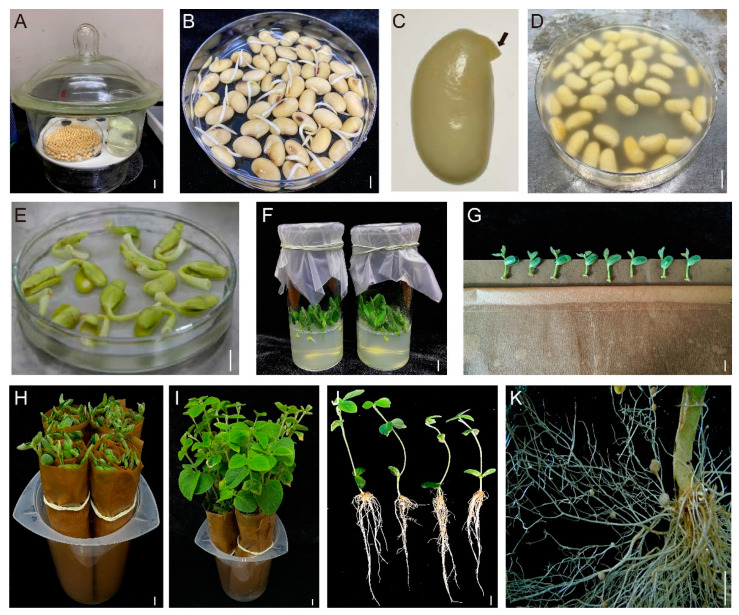
The eR&T workflow in soybean Williams 82. (**A**) Sterilization of soybean seeds. The mature soybean seeds were placed in closed vessels and sterilized with chlorine gas for about 12 h. (**B**) Germinated seeds. Sterile seeds were immersed in sterile water at room temperature to imbibe for about 24 h. (**C**) Explants with approximately 2 mm of hypocotyl for infection. The hypocotyls were trimmed to 2 mm. The black arrow represents the residual hypocotyl (approximately 2 mm). (**D**) *Agrobacterium rhizogenes* infection of 2 mm of hypocotyl explants. The prepared explants were immersed in a suspension of *A. rhizogenes* K599 containing construct of interest (OD_600_ = 0.8–1.0) for 30 min. (**E**) Explants after three days of co-culture. (**F**) Explants growing in HRIM. (**G**) Explants placed on a piece of germination paper. (**H**) Rolls of the explants in a plastic beaker with BD medium. (**I**) Seedlings growing in BD medium at 25 °C with a photoperiod of 16 h light/8 h dark for 10 days. (**J**) Composite plants composed of transgenic roots and wild-type shoots. (**K**) Hairy roots with nodules. The scale bar indicates 1 cm.

To establish an efficient, simple and rapid system of *Agrobacterium rhizogenes*-mediated hairy root transformation, we optimized and integrated the advantages of existing methods of hairy root transformation [5,6,10,11,12,13] namely an efficient regeneration and transformation method of hairy roots (eR&T). In this approach, mature soybean seeds (Williams 82) were placed in closed vessels and sterilized with chlorine gas for about 12 h (Figure 1A). To obtain explant resources for infection, sterile seeds were immersed in sterile water at room temperature, and after one-day germination (Figure 1B), we used a scalpel to remove the seed coat attached to the imbibed seeds and trimmed the hypocotyl to about 2 mm (Figure 1C). Seeds one day after germination with approximately 2 mm of hypocotyl were used as explants for hairy root transformation mediated by *Agrobacterium rhizogenes* (Figure 1C). The prepared explants were immersed in a suspension of *A. rhizogenes* K599 containing the construct of interest (such as gene overexpression or CRISPR/Cas9 gene editing vectors) (OD_600_ = 0.8–1.0) for 30 min (Figure 1D). After infection, explants were placed on a piece of filter paper containing a small amount of liquid co-culture medium (LCCM), and were co-cultured for 3 to 4 days at 23 °C in the dark (Figure 1E). After co-culture, approximately 10 explants were inserted into hairy root induction medium (HRIM) under a photoperiod of 16 h light/8 h dark and a temperature of 23 °C (Figure 1F). After three days, eight elongated explants were rolled up in a piece of germination paper (Figure 1G) to ensure visibility of the explants from the top, and the rolls placed upright in a sterile plastic beaker with BD medium (Figure 1H). Infected explants were allowed to grow in an incubator at 25 °C with a photoperiod of 16 h light/8 h dark for 10 days (Figure 1I,J). Plants with positive hairy roots were selected for subsequent experiments, such as nodulation studies (Figure 1K).

**Figure 2:**
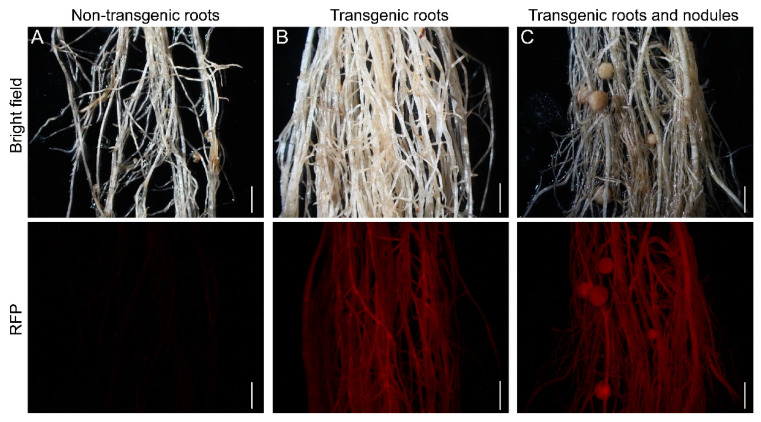
Selection of positive transgenic roots of soybean Williams 82. (**A**) 14-day-old non-transgenic roots. (**B**) 14-day-old transgenic roots. (**C**) Transgenic roots and nodules. Transgenic roots and nodules observed with a stereomicroscope using an RFP filter. Upper panels show bright-field images and the lower panels are epifluorescence microscopy images showing RFP expression in the same roots. The scale bar indicates 5 mm.

**Figure 3:**
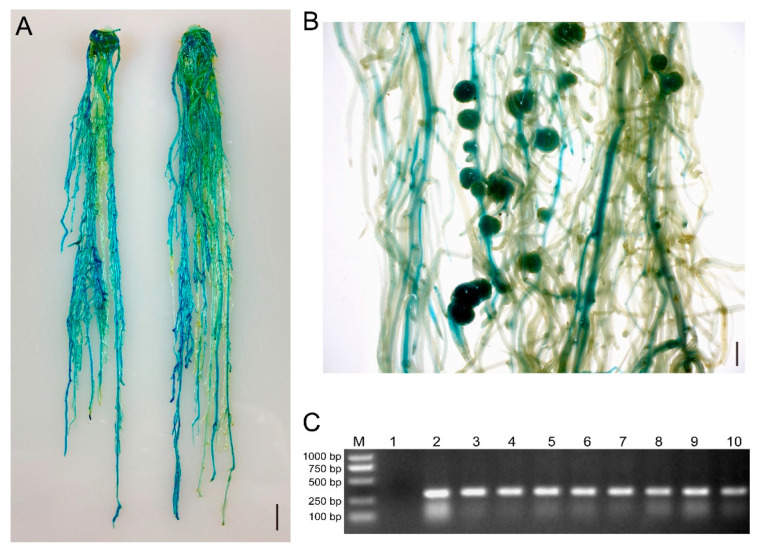
GUS staining and PCR were used to identify positive hairy roots and nodules of soybean Williams82. (**A**) Two representative roots expressing the *GUS* reporter gene. The scale bar indicates 1 cm. (**B**) Representative image of GUS-positive nodules in composite plants at 21 dpi with *S. fredii* HH103. The scale bar indicates 5 mm. (**C**) Gel determination of GUS-positive hairy roots. Lane 1, GUS-negative root; Lane 2–10, GUS-positive root. The transformed hairy roots in (**A**) were subjected to PCR detection using primers of Intron-F and GUS-289R.

Recently, a novel hairy root transformation method was reported in which inoculation involved a thin layer of bacterial paste on the cut surface of shoots/explants rather than dipping the cut surface in bacterial solution [11]. We refer to this approach as a traditional method here, compared to our eR&T. Transgenic roots were identified under a stereomicroscope equipped with an RFP filter (*DsRed2* was the report gene on the binary vector, [14]) (Figure 2 and Supplemental Figure S1). GUS staining was another, positive selective marker, since hairy roots transformed with K599 harbor the *GUS* reporter gene driven by cauliflower mosaic virus (CaMV) 35S promoter. (Figure 3A, B). The third indictor of positive hairy roots was based on PCR analysis of the *GUS* gene (Figure 3C). The three lines of evidence (Figure 2 and Figure 3) were in good agreement with each other, indicating that the eR&T had higher transformation efficiency. Each infected explant had an average of 11.03 ± 2.76 hairy roots, among which there were 7.03 ± 2.66 positive transgenic roots in the eR&T, while in the traditional transformation method, each explant had an average of only 3.88 ± 1.77 hairy roots with 1.35 ± 1.05 positive roots. The transformation frequency of the eR&T reached 63.7%, significantly higher than that of the traditional method (35.8%) (Table 1). Furthermore, the transformation efficiency of the eR&T was almost 100%, while that of the traditional method was only 75% (Table 1), which shows the higher transformation efficiency of the eR&T compared to the traditional method. Additionally, we also compared the transformation efficiency and frequency of two soybean genotypes, Williams 82 and Tianlong 1, and found that Tianlong 1 had a higher transgenic frequency than Williams 82 (70.1% vs. 62.7%), and the transformation efficiency was comparable (97.5% vs. 95%) (Table 2), indicating the wide potential use of the eR&T.

Transformation efficiency indicates the percentage of explants that produced at least one transgenic root in the total transformed explants. Transformation frequency is expressed as a percentage of the number of transgenic roots in the total number of hairy roots. 

### 2.2. Application of eR&T to Promoter Expression Analysis

**Figure 4:**
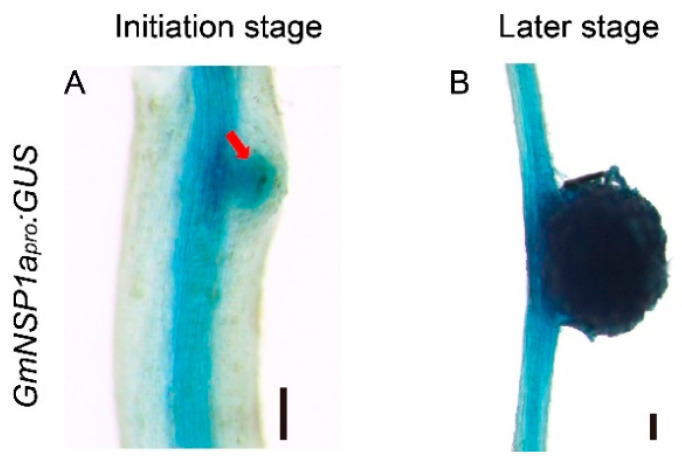
Tissue-specific expressions of *GmNSP1a* in transgenic roots and nodules of soybean Williams82. (**A**,**B**) *GmNSP1a_pro_:GUS* expression pattern during nodulation at 3 dpi (**A**) and 21 dpi (**B**) are shown. The red arrow represents the nodule primordia. The scale bar indicates 200 µm.

Previous results showed that Nodulation Signaling Pathway 1 (*GmNSP1*) is required for rhizobial infection, nodule initiation, and symbiotic gene expression in soybean [15]. The results of GUS staining in hairy roots of *GmNSP1a_pro_:GUS* showed that the promoter activity of *GmNSP1a* is involved at different developmental stages of nodules [15]. To test whether the eR&T was suitable for analyzing the expression pattern of soybean gene promoters, the promoter of the *NSP1a* gene was cloned and marked with the *GUS* reporter gene, and the resulting construct (*GmNSP1a_pro_:GUS*) was integrated with the eR&T, into soybean hairy roots inoculated with *Sinorhizobium fredii* HH103. The hairy roots and nodules were subjected to GUS staining at 3 days post inoculation (dpi) with rhizobia (Figure 4A) and 21 dpi (Figure 4B). Consistent with previous findings, the promoter activity of *GmNSP1a* was expressed in the root stele and nodules at early and late stages of nodule development (Figure 4).

**Figure 5:**
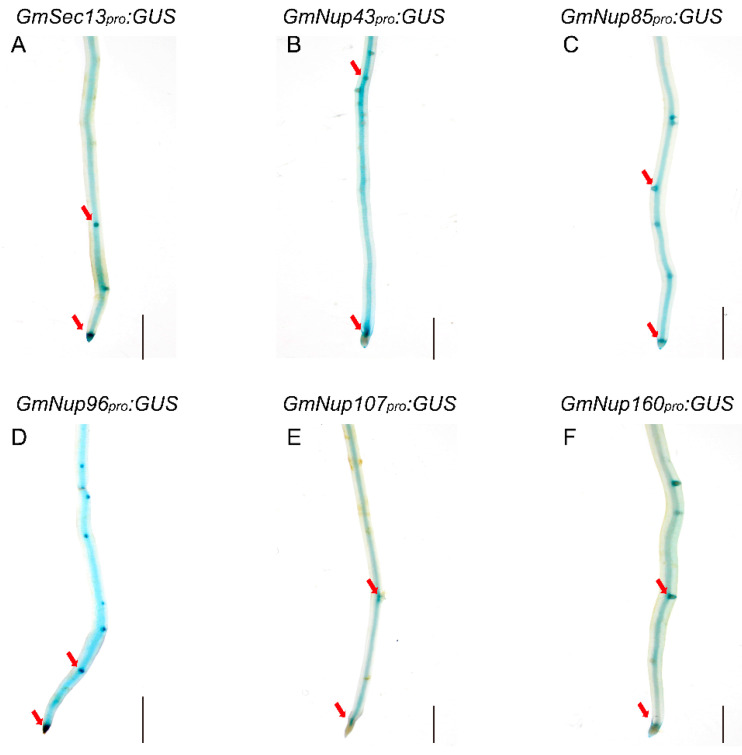
Representative GUS staining in transgenic roots of *GmNup107-160 promoter:GUS* of soybean Williams82. (**A**) *GmSEC13_pro_:GUS*. (**B**) *GmNup43_pro_:GUS*. (**C**) *GmNup85_pro_:GUS*. (**D**) *GmNup96_pro_:GUS*. (**E**) *GmNup107_pro_:GUS*. (**F**) *GmNup160_pro_:GUS*. The red arrow represents the apical and lateral root primordia. The scale bar indicates 5 mm.

**Figure 6:**
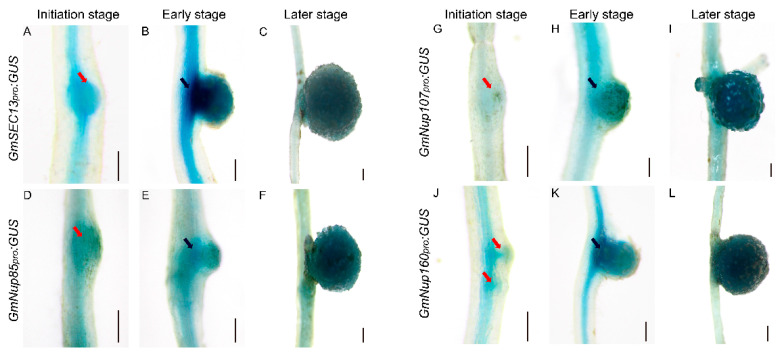
Spatial expression patterns of GmNup107-160 sub-complex promoters during nodulation in soybean Williams82. (**A**–**C**) *GmSEC13_pro_:GUS*. (**D**–**F**) *GmNup85_pro_:GUS*. (**G**–**I**) *GmNup107_pro_:GUS*. (**J**–**L**) *GmNup160_pro_:GUS*. Roots at initial stage (3 dpi) (**A**,**D**,**G**,**J**), at early stage (7 dpi) (**B**,**E**,**H**,**K**), and at later stage (21 dpi) (**C**,**F**,**I**,**L**) are shown. The scale bar indicates 200 µm. The red arrow represents the nodule primordia, the black arrow represents the connective tissues between the root and the nodule.

Previous studies have shown that nucleoporins (Nup85, Nup133, and SEC13) are required for calcium spiking in nodulation that is induced in response to Nod factors in Lotus japonicus [16,17,18]. With curiosity about whether other nucleoporins would participate in the process of nodulation, we focused on outer ring complex—Nup107-160 subcomplex in nuclear pore complex and cloned the promoters of its component genes, including *GmSEC13*, *GmNup43*, *GmNup85*, *GmNup96*, *GmNup107*, and *GmNup160*, and marked with the *GUS* gene as *GmNSP1a* promoter (Figure 4) to investigate their expression patterns in roots and nodules. GUS staining showed that promoters of these genes shared similar expression patterns in the roots, with stronger signals in apical and lateral root primordia (Figure 5 and Supplemental Figure S2). Similarly, these promoters were also expressed at different stages of nodule development (Figure 6 and Supplemental Figure S3). However, the different promoters expressed obvious differences. For example, the promoter activity of *GmSEC13* was expressed in different stages and nodule tissues, but with higher signals in tissues connecting the root and the nodule at the early stage of nodule development (Figure 6B). The promoter activity of *GmNup85*, *GmNup107*, and *GmNup160* was increased along with the nodule development process (Figure 6D–L). These results suggest that these nucleoporin components may have different functions during nodule development, even though they share similar processes. Therefore, the eR&T is reliable and feasible for analyzing the expression patterns of soybean gene promoters.

### 2.3. Application of the eR&T for GmNSP1a:GFP Subcellular Localization in Soybean

**Figure 7:**
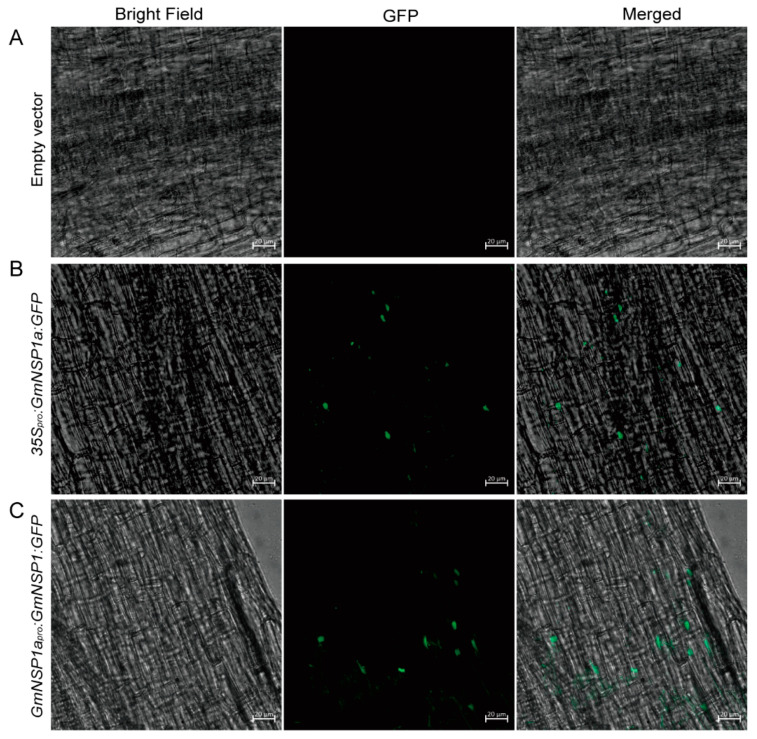
Subcellular localization of GmNSP1a:GFP fusion proteins in soybean Williams82. Soybean Williams82 transformed with *35S_pro_:GmNSP1a:GFP* (**B**) and *GmNSP1a_pro_:GmNSP1a:GFP* (**C**) in hairy roots. Non-transgenic roots were used as a negative control (**A**). Fluorescence was detected by confocal microscopy. The green spots represent nuclei. The scale bar indicates 20 µm.

The correct subcellular localization of proteins is a prerequisite for their normal functions. An exogenous transient expression system, such as *Nicotiana benthamiana* leaves and *Arabidopsis* leaf protoplasts, cannot reflect the correct or native localization of soybean proteins in soybean cells. Therefore, *Agrobacterium rhizogenes*-mediated hairy root transformation provides a suitable method for studying the subcellular localization of proteins in soybean cells. *NSP1* encodes a GRAS family protein that is localized in nuclei to bind to the promoter of the Nod factor inducible genes *ENOD11*, *ERN1*, and *NIN* [15,19,20]. As show in Figure 7, GmNSP1a:GFP fusion proteins driven by *35S* or *GmNSP1a* promoters, respectively, were localized in the nuclei of hairy root cells. These results indicate that the eR&T provides a suitable method for studying the subcellular localization of proteins in soybean cells.

### 2.4. Application the eR&T for Generating CRISPR/Cas9 Mutants

**Figure 8:**
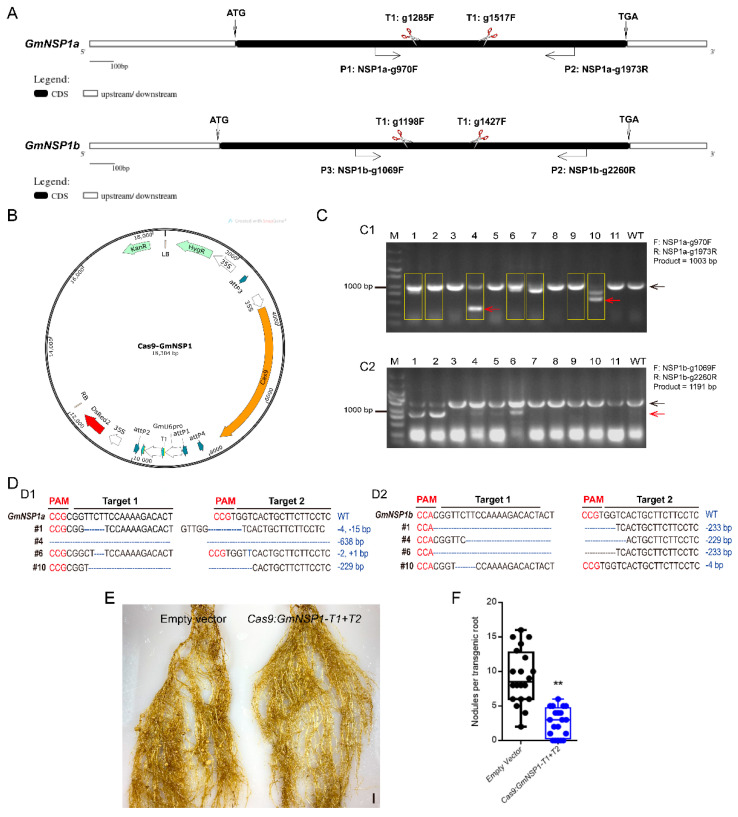
Generation of *Gmnsp1a Gmnsp1b* double mutants by CRISPR/CAS9 in soybean Williams82 hairy roots. (**A**) Schematic structures of the *GmNSP1a* and *GmNSP1b* genes marked with target sites of sgRNAs and detecting primers. (**B**) Map of the binary vector for *GmNSP1a/b* gene−editing. (**C**) Gel determination of *GmNSP1a* (**C1**) and *GmNSP1b* (**C2**) gene-edited hairy roots. PCR detection using primers of (GmNSP1a-g970F + GmNSP1a-g1973R) or (GmNSP1b-g1069F + GmNSP1b-g2260R). The yellow box represents *GmNSP1a* gene−edited hairy roots. (The black arrow represents the big PCR bands and the red arrow represents the small PCR bands). (**D**) Sequencing determination of *GmNSP1a* and *GmNSP1b* gene edited roots (#1, #4, #6, and #10). (**D1**) the total PCR products (#1, #6) or small PCR bands (red arrow; #4, #10) in Figure (**C1**) were cloned into pGWC vector [22] for transforming into *E. coli* DH5α for sequencing. (**D2**) Total PCR products (#10) or small PCR bands (red arrow; #1, #4, and #6) in Figure (**C2**) were cloned into pGWC vector for transforming into *E. coli* DH5α for sequencing. (**E**) Representative images of nodule phenotypes in empty vector and the *Gmnsp1a Gmnsp1b* double mutants at 21 dpi with *S. fredii* HH103. (**F**) Statistical analysis of the number of nodules on gene−editing roots, n = 20. Significant at Student’s *t*-test. ** (*p* < 0.01).

Next, we investigated the efficiency of the eR&T in constructing soybean mutants mediated by CRISPR/Cas9 module using two nodule-regulating genes, *GmNSP1a* and *GmNSP1b* [15], as target genes. We designed two individual sgRNAs for two target genes (Figure 8A) on the Web-based tool CRISPR-P (http://crispr.hzau.edu.cn/cgibin/CRISPR2/CRISPR; accessed on 8 July 2020 [21]). The binary vector bearing intact expression frames of both Cas9 and sgRNA (Figure 8B) was introduced into *A. rhizogenes* K599 for hairy root transformation, and then positive hairy roots with DsRed2 fluorescence (as in Figure 2) were selected for further mutant identification by PCR (Supplemental Table S1). PCR results showed that there was a smaller extra band in some positive hairy roots compared to WT in a gel, as indicated by the red arrow in Figure 8C. Sequencing data of PCR products (#1, 2, 4, 6, 7, 9, and 10) in Figure 8C1 shows chaotic peaks near the Target 1 sequence of *GmNSP1a* compared to wild type (Supplemental Figure S4 and Supplemental data), indicating that these roots may be edited (yellow box in Figure 8C). Further cloning and sequencing these bands in Figure 8C1 (#1 and #6, big bands; #4 and #10, small bands) and Figure 8C2 (#10, big bands; #1, #4, and #6; small bands) indicated that *GmNSP1a* (Figure 8D1) and *GmNSP1b* (Figure 8D2) of these hairy roots were successfully edited. These mutants were subjected to induce nodulation mediated by *S. fredii* HH103, and they produced less nodules than wild type roots (Figure 8E,F), consistent with previous reports [15,19]. These results indicate that the eR&T is suitable for producing CRISPR/Cas9 mutants.

## 3. Discussion

### 3.1. An Efficient Method for Hairy Roots Regeneration and Transformation in Soybean

With easy operation and short duration, hairy root generation and transformation is a practical method, especially in root and nodule biology, for the study of various plants, including soybean [5,6,11,12]. Therefore, several methods of hairy root transformation were reported, and each of them has its own advantages and disadvantages. For example, the Song et al. method [11] is simple and time-saving, but the transformation efficiency (75%) and transformation frequency (35.8%) are relatively low compared to our experimental method. Cheng et al. [12] attained a higher efficiency of transformation, but the positive hairy roots were not suitable for the subsequent nodulation analysis, since the researchers used a 2/3 portions of cotyledon without embryos as explants, which could not produce shoots later in development. Obviously, there is still much to do to improve hairy root transformation. We integrated and optimized current approaches to develop a new method eR&T, and verified its practicability with a series of experiments. Compared to current approaches, the eR&T stands out with its efficiency of both regeneration and transformation of hairy roots in intact composite plants with healthy shoots. Compared to the previous hairy root transformation [11] methods, the eR&T has a higher regeneration ratio (11.03 ± 2.76 vs. 3.88 ± 1.77), transformation efficiency (100% vs. 75%) and transformation frequency (63.7% vs. 35.8%) (Table 1). A larger number of roots from a single plant is good for intra-batch comparisons. Additionally, in our experience, one person is able to perform transformation of up to 200 seeds in one round of experiments, and so many seeds can be divided into 10 individual experiments with 20 replicates each, beneficial for both batch comparison experiments and saving space. It takes only 18 to 20 days for one round of the eR&T (Figure 1), conferring the eR&T the advantage of saving time. All steps in the eR&T are carried out in aseptic conditions or semi-aseptic conditions, which reduces interference from microorganisms in the soil and allows experiments with controlled conditions, such as different nutrients, for analysis of root and nodule biology. The hairy roots and nodules from the eR&T are not interwoven like those of soil-based growth, but separated, which helps root and nodule morphological analysis.

Additionally, since hairy roots are widely employed in many plants, especially species recalcitrant to stable genetic transformation, such as *Rehmannia glutinosa* [23], *Panax ginseng* [24], *Cicer arietinum* [25], *Trachyspermum ammi* [26], *Rubia yunnanensis* [27], *Taxus* spp. [28], *Trigonella foenum-graecum* [29], the eR&T is expected to be modified for application in these plants. For example, in vitro cultures of plants, including hairy roots, have been widely used in biotechnology to obtain high-value plant-derived products with a variety of biological and pharmacological activities [30,31,32]. Despite great progress in biotechnological approaches to paclitaxel production, the productivity of *Taxus* spp. in vitro cultures remains a challenge [28]. Paclitaxel has become a widely used anticancer drug for the treatment of various malignancies, including breast and ovarian cancers and Kaposi’s sarcoma [33,34]. Fenugreek, a non-model crop legume with important medicinal value, produces a variety of specialized metabolites, such as diosgenin [29,35]. In the light of the efficiency, simplicity and economy of the eR&T, we expect that the eR&T may be widely used to obtain high-value plant-derived products with a variety of biological and pharmacological activities.

### 3.2. The eR&T Is Reliable and Feasible to Gene Functional Analysis

As well as biological analysis of roots and nodules, hairy roots are widely used in functional study of genes or regulating elements such as promoters [36,37,38,39], providing species-specific or native data for crop plants other than heterologous or non-native evidence from model plants such as *Arabidopsis* or *Nicotiana benthamiana* leaves. The eR&T, developed by our study was evaluated and verified in a series of experiments with genes and promoters, including expression pattern of promotors, protein subcellular localization, and generation of CRISPR/Cas9-edited mutants. The results of known genes in this study are consistent with previous reports, such as Gm*NSP1a* expression pattern (Figure 4) [15,19] and its function in nodulation (Figure 8) [15,19] and Gm*SEC13* expression pattern in nodules (Figure 6) [16,17,18]. With the eR&T, we also identified novel potential regulators of root development and nodulation, such as *GmNup43*, *GmNup96*, *GmNup107*, and *GmNup160* (Figure 5, Figure 6, Figures S2 and S3). GUS staining showed that promoters of these genes shared similar expression patterns in the roots, with stronger signals in apical and lateral root primordia (Figure 5 and Supplemental Figure S2). These promoters were also expressed at different stages of nodule development (Figure 6 and Supplemental Figure S3). However, differences in the expressions of different promoters were also evident. The promoter activity of *GmSEC13* was expressed at different stages and in different tissues of nodules, but with higher signals in tissues connecting the root and the nodule at early stages of nodule development (Figure 6B). The promoter activity of *GmNup85*, *GmNup107*, and *GmNup160* increased along with the nodule development process (Figure 6D–L), indicating that these genes are required for nodule development. The results suggest that GmNup107 and GmNup160 might be novel nodule-developmental regulators such as GmNup85 than GmSEC13 do [17,18]. It would be interesting to use the eR&T to investigate the interaction of individual nucleoporins in the Nup107-160 subcomplex in nodulation.

The CRISPR/Cas9 system is a powerful gene editing tool for the generation of gene mutations [12,40,41]. However, generating new mutants using the CRISPR/Cas9 system is a time-consuming process for most plants, including soybean. In addition, inappropriate selection of sgRNA may lead to the risk of off-target or low efficiency. Benefiting from its time and space saving characteristics and efficiency, the eR&T is expected to be widely applied in verifying sgRNA targets of CRISPR/Cas9 gene editing before starting stable genetic transformation. The eR&T could also be used for the functional investigation of genes or promoters of interest, such as protein-protein interaction and transcriptional activity of a promoter regulated by a transcription factor.

## 4. Materials and Methods

### 4.1. Plant Materials and Growth Conditions

Soybean seeds of two soybean genotypes Williams 82 and Tianlong 1 were used in this study. Soybean seeds were surface-sterilized for 6–12 h with chlorine gas (100 mL NaClO + 3.5 mL HCl) in a tightly sealed chamber (Figure 1A) [4]. Sterile seeds were immersed in sterile water at room temperature to imbibe for 24 h to prepare explants for transformation. Plants were incubated in a sterile plastic beaker with BD medium (Figure 1H,I) and grown in plant growth chamber under 80 μmol m^−2^ s^−1^ for 25 °C with a photoperiod of 16 h light/8 h dark, and at approximately 80% relative humidity. For the nodulation assays, plants with positive transgenic roots were transplanted into pots with completely wet sterile vermiculite (12 cm × 8 cm), which was watered with BD medium. Plants were grown in a greenhouse under 120 μmol m^−2^ s^−1^ for 25 °C with a photoperiod of 16 h light/8 h dark.

### 4.2. Vector Constructions and A. rhizogenes Strain

For promoter analysis, we cloned the promoter sequence approximately 2.0 kb upstream of the translational start code (ATG) of *GmSEC13a* (Glyma.08G321800), *GmNup43a* (Glyma.17G071100), *GmNup85* (Glyma.17G193800), *GmNup96* (Glyma14g00880), *GmNup107* (Glyma.07G042000), *GmNup160* (Glyma.10G055100), and *GmNSP1a* (Glyma.16G008200), and individually cloned into the vector Fu76 [42]; the *GUS* reporter was introduced into the entry vector Fu79 [42]. Then, through the LR reaction (Invitrogen, Carlsbad, CA, USA) these entry clones were combined into pSoy10 with attR1/2 and attR3/4 recombination sites, which were modified from pHairyRED [14] to generate expressing constructs. For gene editing in soybean, we constructed two entry vectors to express Cas9 or double-guide RNAs (sgRNAs). A Cas9 gene [43] driven by the *CaMV 35S* promoter was cloned into vector Fu76, and the sgRNA scaffold were cloned into vector Fu79. To design sgRNA of target genes, we used the Web-based tool CRISPR-P (http://crispr.hzau.edu.cn/cgibin/CRISPR2/CRISPR, accessed on 8 July 2020) [21]. Two target sequences were synthesized and cloned into Fu79-sgRNA with *Bsa*I and *Bsp*QI, respectively. Finally, Fu76-Cas9 and Fu79-sgRNA were combined into pSoy10 to generate binary vectors for gene editing (Figure 8B). All the resulting expression vectors above were introduced into *A. rhizogenes* strain K599 and were used to induce hairy roots [44]. All primers used in this paper are shown in Supplemental Table S1.

### 4.3. Optimized Agrobacterium rhizogenes-Mediated Hairy Root Transformation

*Agrobacterium rhizogenes*-mediated hairy root transformations were performed as described previously, with modifications [4,12,45]. Briefly, a single colony of *A. rhizogenes* strain K599 harboring a gene construct of interest from the plate was inoculated in a tube containing 4 mL liquid YEB with 50 mg/L kanamycin for selection and cultured for 12 h at 28 °C (200 rpm) to obtain the starter culture. On the next day, 200 μL of the starter culture was transferred to 100 mL YEB culture with 50 mg/L kanamycin and incubated at 28 °C (200 rpm) in a shaker incubator until the OD_600_ reached 1.0. The *Agrobacterium rhizogenes* culture was collected at 2000× *g* for 10 min, and then the pellet was resuspended in liquid co-cultivation medium (LCCM, 1/10X B5 basal medium supplemented with 3.9 g/L MES, 3% (30 g/L) sucrose, pH 5.4 (adjusted with KOH), 40 mg/L acetosyringone (AS), and DTT (154.2 mg/L)) to an OD_600_ of 0.8–1.0.

The seed coat and roots were removed, and explants with about 2 mm of hypocotyl (Figure 1C) were put in a suspension of the *A. rhizogenes* strain K599 harboring the construct of interest (Figure 1D) for 30 min. After infection, 15 to 20 explants were placed on sterile filter paper to air dry briefly, then the explants were placed on a piece of filter paper wetted with LCCM in advance and cultured for three days (Figure 1E). After co-cultivation, the explants were inserted into hairy root induction medium (HRIM, 1X B5 basal medium, 0.59 g/L MES, 3% (30 g/L) sucrose, 0.7% (7 g/L) agarose, pH = 5.7 (adjusting by KOH), 100 mg/L Timentin, 100 mg/L Cefotaxime). After three days, explants with elongated stems and expanded hypocotyls were selected. Then, the infected explants were rolled up in a piece of moist germination paper (38*25 cm; Anchor Paper Co., St. Paul, MN, USA) and incubated for 10 days in a sterile plastic beaker with BD medium (Figure 1G–I).

### 4.4. GUS Staining

Histochemical analysis of GUS expression was performed according to a previous report [45,46] with minor modifications. We selected positive transgenic hairy roots with an RFP fluorescence marker using the pHairyRED binary vector [14]. The positive transgenic hairy roots and nodules were then soaked in 90% (*v*/*v*) acetone for 30 min for fixation and incubated in X-Gluc solution (50 mM sodium phosphate buffer, 0.2% Triton X-100, 5 mM K_4_Fe(CN)_6_, 5 mM K_3_Fe(CN)_6_, and 1–2 mM X-gluc) overnight at 37 °C. Then, samples were transferred through an ethanol series (20, 30, 50, and 70% ethanol) and examined with a microscope.

### 4.5. Subcellular Localization

The coding sequence of *GmNSP1a* was introduced into the entry vector Fu28 [42], and the 2.0-kb genomic sequence upstream of the start codon was cloned into Fu76 resulting in a *GmNSP1a* promoter entry clone. The resulting entry clones were combined into pSoy10 to generate *GmNSP1a_pro_:GmNSP1a:GFP* or *35S_pro_:GmNSP1a:GFP* binary vectors by LR reaction (Invitrogen). Transgenic hairy roots were prepared for fluorescence observations under a Zeiss LSM980 confocal laser scanning microscope.

### 4.6. Soybean Nodulation

A nodulation assay was performed according to a previous report [45]. Successful transformation was indicated by the presence of fluorescence from RFP, which was used as a visual marker as for roots described above. Three positive transgenic roots were retained from each plant. For the nodulation assays, the plants with positive roots were transplanted into pots with completely wet sterile vermiculite (12 cm × 8 cm) watered with BD medium [6] (i.e., 500 mL of each stock solution per liter: stock solution A (2 M CaCl_2_), stock solution B (1 M KH_2_PO_4_), stock solution C (20 mM Fe-citrate), stock solution D (0.5 M MgSO_4_, 0.5 M K_2_SO_4_, 2 mM MnSO_4_, 4 mM H_3_BO_4_, 1 mM ZnSO_4_, 4 mM CuSO_4_, 0.2 mM CoSO_4_, 0.2 mM Na_2_MoO_4_)) and grown in the dark for 3–4 days to allow them to acclimate to the environment. The acclimated seedlings were inoculated with *S. fredii* HH103 (OD_600_ = 0.1; 30 mL). After 3–4 weeks, the phenotypes of nodules were investigated. Genotyping of edited hairy roots was carried out by PCR with a pair of primers covering two CRISPR/Cas9 target sites (Figure 8 and Supplemental Table S1).

### 4.7. Statistical Analysis

Each experiment with 20 seedlings represented a biological replicate, and all experiments had three biological replicates with consistent results. Statistical analysis was carried out using the SPSS software package (https://www.ibm.com/analytics/spssstatistics-software, accessed on 15 March 2022). Asterisks indicate significant differences according to Student’s *t*-test (**, *p* < 0.01). Means with letter a and b denotes a significance difference by One-way analysis of variance (*p* < 0.01).

## 5. Conclusions

We established a practical and efficient transformation system (the e&RT) mediated by *Agrobacterium rhizogenes* to generate a large number of composite plants with many hairy roots in soybean, which is suitable for investigation of root and nodule biology, gene and promoter functions, and mutant generation in hairy roots.

## Figures and Tables

**Table 1:** Comparison of transformation efficiency and frequency between the traditional method and the eR&T in Williams82.

Method	Total Number of Explants	Number of Explants with Hairy Roots	Average Number of Hairy Roots per Explant	Average Number of GUS Positive Roots per Explant	Transformation Efficiency	Transformation Frequency
Traditional method	40	30	3.88 ± 1.77 ^b^	1.35 ± 1.05 ^b^	75%	35.8%
eR&T method	40	40	11.03 ± 2.76 ^a^	7.03 ± 2.66 ^a^	100%	63.7%

Means with letter ^a^ and ^b^ denote a significance difference (ANOVA test, *p* < 0.01).

**Table 2:** Comparison of transformation efficiency and frequency of the eR&T between Williams82 and Tianlong1.

Genotype	Total Number of Explants	Number of Explants with Hairy Roots	Average Number of Hairy Roots per Explant	Average Number of GUS Positive Roots per Explant	Transformation Efficiency	Transformation Frequency
Williams 82	40	38	10.45 ± 3.52 ^a^	6.55 ± 3.34 ^b^	95%	62.7%
Tianlong 1	40	39	10.55 ± 2.97 ^a^	7.40 ± 2.99 ^a^	97.5%	70.1%

Note: See Table 1. Means with letter ^a^ and ^b^ denote a significance difference (ANOVA test, *p* < 0.01).

## Data Availability

Data are contained within the article and Supplementary Materials.

## Supplementary Materials

The following supporting information can be downloaded at: https://www.mdpi.com/article/10.3390/ijms232012261/s1.
