# Supplementary material for: An Efficient Agrobacterium rhizogenes-Mediated Hairy Root Transformation Method in a Soybean Root Biology Study
Source: Int J Mol Sci. 2022 Oct 14;23(20):12261. doi: 10.3390/ijms232012261 (PMC9603872; doi:10.3390/ijms232012261)
Supplement: Supplementary file 1 [file ijms-23-12261-s001.zip › Supplemental Figures-IJMS.pdf]

An efficient *Agrobacterium rhizogenes*-mediated hairy root transformation method in soybean root biology study

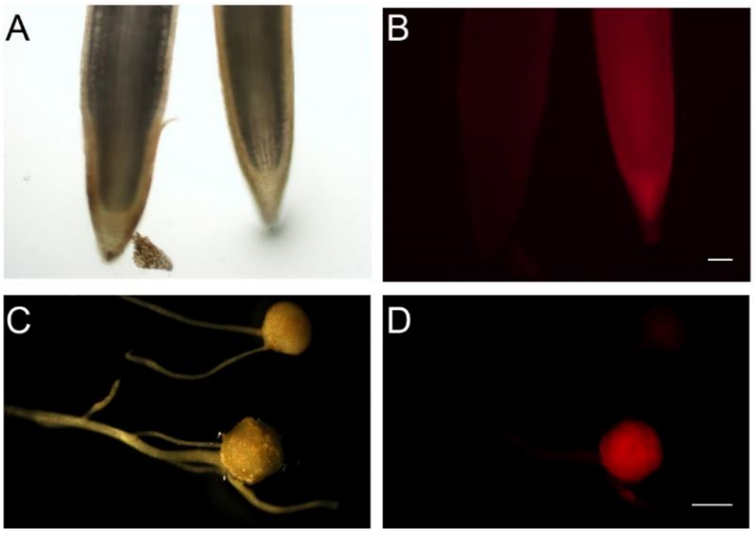

Supplemental Figure S1. Selection of positive transgenic roots of soybean Tianlong1. (A and B) 14-day-old non-transgenic and transgenic roots. (C and D) Non-transgenic and transgenic nodules. Transgenic roots and nodules observed with a stereomicroscope using an RFP filter. (A and C) represent bright-field images, and the (B and D) represent fluorescence images. The scale bar indicates 1 mm.

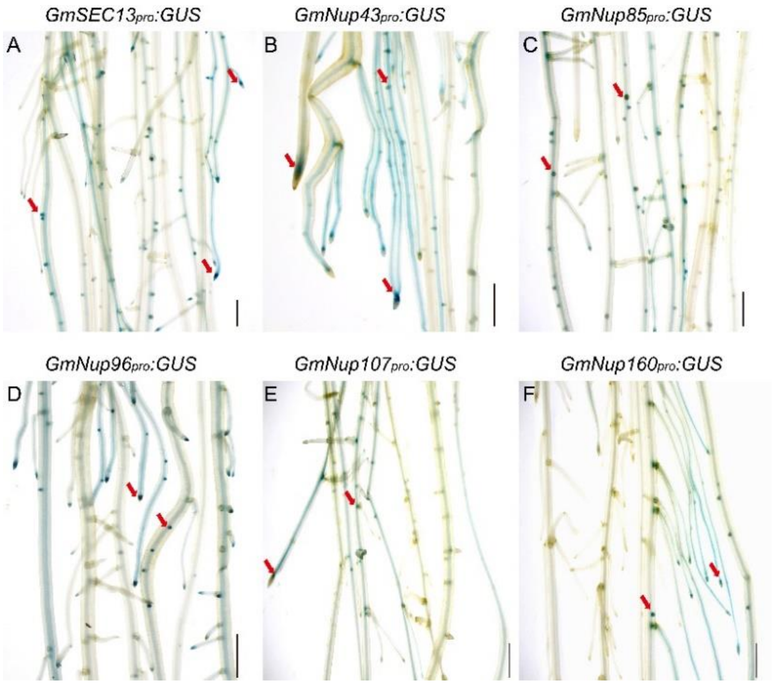

Supplemental Figure S2. Tissue-specific promoter expressions of GmNup107-160 sub-complex components in transgenic roots of soybean Williams 82. (A) *GmSEC13$_{pro}$:GUS*. (B) *GmNup43$_{pro}$:GUS*. (C) *GmNup85$_{pro}$:GUS*. (D) *GmNup96$_{pro}$:GUS*. (E) *GmNup107$_{pro}$:GUS*. (F) *GmNup160$_{pro}$:GUS*. The red arrow represents the apical and lateral root primordia. The scale bar indicates 1 cm.

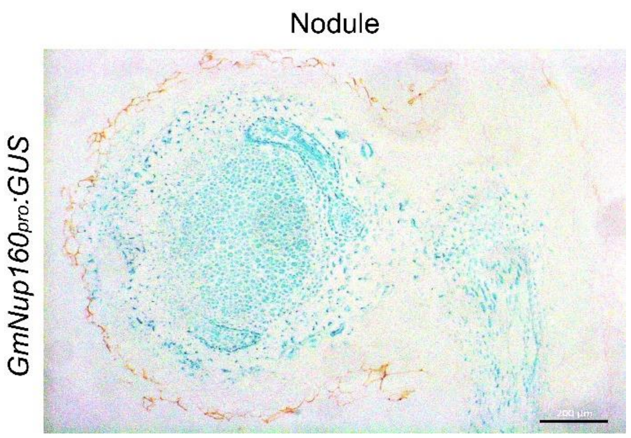

Supplemental Figure S3. Tissue-specific expression of the promoter of *GmNup160* in soybean nodules. Longitudinal sections of GUS stained nodule in *GmNup160Pro:GUS*. Nodules on soybean roots at 14 d after infection were used for GUS staining. The scale bar indicates 200 μm.

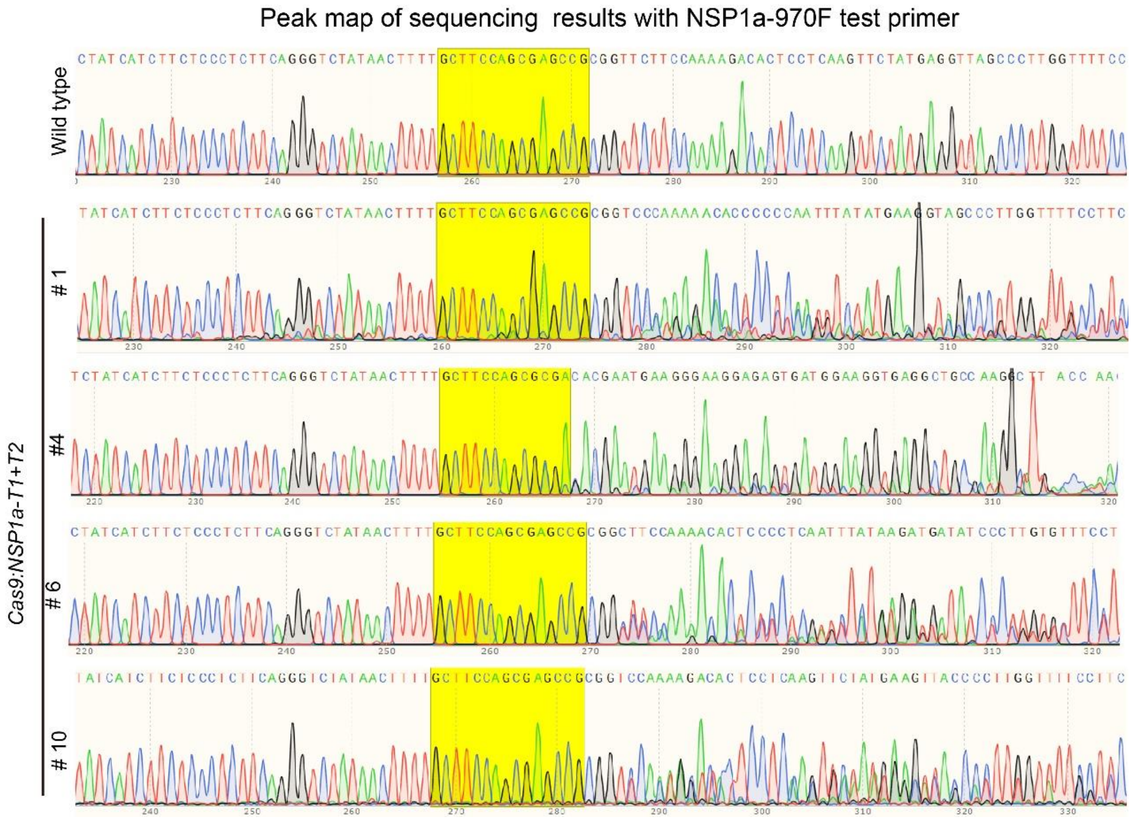

Supplemental Figure S4. Sequencing determination of fragments indicating by a yellow box in (Fig. 8C1) in edited-hairy roots (#1, #4, #6, and #10) using primers of GmNSP1a-970F, showing that the nucleotide residue peaks appear chaotic compared to wild type *GmNSP1a* after partial Target 1 sequence (in yellow background).
