# Supplementary material for: An Efficient Agrobacterium rhizogenes-Mediated Hairy Root Transformation Method in a Soybean Root Biology Study
Source: Int J Mol Sci. 2022 Oct 14;23(20):12261. doi: 10.3390/ijms232012261 (PMC9603872; doi:10.3390/ijms232012261)
Supplement: Supplementary file 1 [file ijms-23-12261-s001.zip › Supplemental Table S1 The list of primers-IJMS.pdf]

Supplemental Table S1 A list of primers used in this study

Primer Name	Sequence (5'-3')	Assay
GmNSP1a$_{pro}$-*Sac*I-F<br>GmNSP1a$_{pro}$-*Spe*I-R	CCGGAGCTCGTAAAAGGGGTAAAAACTTG<br>CCGACTAGTTGTAAATGGAAGCAAGTGTTG	promoter<br>cloning
GmNup43a$_{pro}$-*Xho*I-F<br>GmNup43a$_{pro}$-*Sma*I-R	CCGCTCGAGTTCTACGGATGGTCACTTG<br>CCGCCCGGGGCATAAGAGAACTTAATTCC	promoter<br>cloning
GmNup85$_{pro}$-*Xho*I-F<br>GmNup85$_{pro}$-*Sma*I-R	CCGCTCGAGATTGAATTATCATCTTTAAA<br>CCGCCCGGTTACGCGGGAAATAAATAAA	promoter<br>cloning
GmNup96$_{pro}$-*Sac*I-F<br>GmNup96$_{pro}$-*Spe*I-R	CCGGAGCTCTTCGTCCAGACTTGTTAT<br>CCGACTAGTCACAAAGCTAGGTCAAAGAG	promoter<br>cloning
GmNup107$_{pro}$-*Xho*I-F<br>GmNup107$_{pro}$-*Sma*I-R	CCGCTCGAGGAAGTGCAAACTCATTATTT<br>CCGCCCGGTGGAATGCTTGCATTTTTAAG	promoter<br>cloning
GmNup160$_{pro}$-*Xho*I-F<br>GmNup160$_{pro}$-*Sma*I-R	CCGCTCGAGCAGACAAATTTGAAAATGA<br>CCGCCCGGCAACATTATTCTTTATTTTCAC	promoter<br>cloning
GmSEC13a$_{pro}$-*Xho*I-F<br>GmSEC13a$_{pro}$-*Sma*I-R	CCGCTCGAGTAAACATAGACTAAACGAAT<br>CCGCCCGGAGAGAAAAAAAAGAAATGTTAC	promoter<br>cloning
GmNSP1a-*Eco*RI-F<br>GmNSP1a-*Xho*I-R	CCGGAATTCATGATCATGGAACCAAATC<br>CCGCTCGAGTGATGTAAATGTTGAGGTC	Gene clone
Intron-F<br>GUS-289R	TGACAATGAATCGTGATCGG<br>AAGGCACGATAATGGTCTTCC	genotyping
GmNSP1a-g970F<br>GmNSP1a-1973R	GGACACGCAGAAGGGACAAC<br>CTGGTTTGTCAATGCCTTCG	Genotyping
GmNSP1b-g1069F<br>GmNSP1b-g2260R	GCGACAACAACAACAACAGC<br>GAGAAATTTGGCAAGGAAGAG	Genotyping
GmNSP1-Target1-F<br>GmNSP1-Target1-R	GATTAGTGTCTTTTGGAAGAACCG<br>AAACCGGTTCTTCCAAAAGACACT	CRISPR/CAS9
GmNSP1-Target2-F<br>GmNSP1-Target2-R	ATTGGAGGAAGAAGCAGTGACCA<br>AACTGGTCACTGCTTCTTCCTCC	CRISPR/CAS9
